# Racial/Ethnic Disparity in Association Between Fetal Alcohol Syndrome and Alcohol Intake During Pregnancy: Multisite Retrospective Cohort Study

**DOI:** 10.2196/45358

**Published:** 2023-04-21

**Authors:** Sarah Soyeon Oh, Bada Kang, Jewel Park, SangMin Kim, Eun-Cheol Park, Seung Hee Lee, Ichiro Kawachi

**Affiliations:** 1 Department of Social and Behavioral Sciences Harvard TH Chan School of Public Health Boston, MA United States; 2 Institute of Health Services Research Yonsei University College of Medicine Seoul Republic of Korea; 3 Mo-Im Kim Nursing Research Institute Yonsei University College of Nursing Seoul Republic of Korea; 4 Department of Pediatrics Cincinnati Children’s Hospital Medical Center Cincinnati, OH United States; 5 Harvard Medical School Boston, MA United States; 6 Department of Preventive Medicine and Public Health Yonsei University College of Medicine Seoul Republic of Korea; 7 College of Nursing and Brain Korea 21 Four Project Yonsei University Seoul Republic of Korea

**Keywords:** fetal alcohol syndrome, ethnic disparity, alcohol intake, pregnancy, health disparity, public health, minority population, vulnerable population, women's health, pediatrics, fetal health, pediatrics

## Abstract

**Background:**

Alcohol consumption during pregnancy is associated with a range of adverse birth-related outcomes, including stillbirth, low birth weight, preterm birth, and fetal alcohol syndrome (FAS). With more than 10% of women consuming alcohol during pregnancy worldwide, it is increasingly important to understand how racial/ethnic variations affect FAS onset. However, whether race and ethnicity inform FAS risk assessment when daily ethanol intake is controlled for remains unknown.

**Objective:**

This study aimed to assess racial/ethnic disparities in FAS risk associated with alcohol consumption during pregnancy.

**Methods:**

We used data from a longitudinal cohort study (the Collaborative Initiative on Fetal Alcohol Spectrum Disorders) at 5 hospital sites around the United States of 595 women who consumed alcohol during pregnancy from 2007 to 2017. Questionnaires, in-person interviews, and reviews of medical, legal, and social service records were used to gather data on average alcoholic content (AAC) during pregnancy. Self-reports of maternal race (American Indian/Alaska Native [AI/AN], Asian, Native Hawaiian or other Pacific Islander, Black or African American, White, more than one race, and other) and ethnicity (Hispanic/Latino or not Hispanic/Latino), as well as FAS diagnoses based on standardized dysmorphological criteria, were used for analysis. Log-binomial regression was used to examine the risk of FAS associated with each 1-gram increase in ethanol consumption during pregnancy, stratified by race/ethnicity.

**Results:**

A total of 3.4% (20/595) of women who reported consuming alcohol during pregnancy gave birth to a baby with FAS. Women who gave birth to a baby with FAS had a mean AAC of 32.06 (SD 9.09) grams, which was higher than that of women who did not give birth to a baby with FAS (mean 12.07, SD 15.87 grams). AI/AN mothers with FAS babies had the highest AAC (mean 42.62, SD 8.35 grams), followed by White (mean 30.13, SD 4.88 grams) and Black mothers (mean 27.05, SD 12.78 grams). White (prevalence ratio [PR] 1.10, 95% CI 1.03-1.19), Black (PR 1.13, 95% CI 1.04-1.23), and AI/AN (PR 1.10, 95% CI 1.00-1.21) mothers had 10% to 13% increased odds of giving birth to a baby with FAS given the same exposure to alcohol during pregnancy. Regardless of race, a 1-gram increase in AAC resulted in a 4% increase (PR 1.04, 95% CI 1.02-1.07) in the chance of giving birth to a baby with ≥2 facial anomalies (ie, short palpebral fissures, thin vermilion border of the upper lip, and smooth philtrum) and a 4% increase (PR 1.04, 95% CI 1.01-1.07) in the chance of deficient brain growth.

**Conclusions:**

The risk of delivering a baby with FAS was comparable among White, Black, and AI/AN women at similar levels of drinking during pregnancy. Regardless of race, a 1-gram increase in AAC resulted in increased odds of giving birth to a baby with facial anomalies or deficient brain growth.

## Introduction

Alcohol consumption during pregnancy is associated with a range of adverse birth-related outcomes, including stillbirth, low birth weight, preterm birth, and fetal alcohol spectrum disorders (FASDs) [1]. FASDs range from physical defects to cognitive, social, behavioral, and emotional impairments [2]. Fetal alcohol syndrome (FAS) is the most severe form of FASDs, which are defined as a range of lifelong congenital anomalies, pre- or postnatal growth restrictions, and dysmorphic facial features originating from prenatal alcohol exposure [3].

Globally, around 10% of women consume alcohol during pregnancy, of whom 1 in 13 will deliver a child with a FASD, and 1 in 67, a child with FAS [4]. Previous studies have reported racial/ethnic variations in alcohol consumption during pregnancy [4]. Black, Asian/Pacific Islander, and Hispanic women drink less than White women during pregnancy [5], while non-Hispanic Black women are up to 48% and 62% less likely to drink alcohol during pregnancy and binge drink, respectively, compared to non-Hispanic White women [6]. Controlling for socioeconomic background, social support, paternal health behaviors, and maternal medical history, Black and Hispanic mothers are 41% and 58% less likely to drink during pregnancy, respectively, compared to White mothers [7].

However, although White women may drink more alcohol than Black/African American or Hispanic women before and during pregnancy [8], they are less likely than their counterparts to be at risk of adverse birth-related outcomes [9]. The fetal mortality rate among Black women (11.2%) is nearly twice that of White (5.9%) and Hispanic (5.1%) women in the United States [9]. Black women are also known to be at greater risk of both unintended pregnancies and poor pregnancy outcomes, such as preterm birth or low birth weight [10].

Concerning FAS and FASDs, Black infants have been reported to have a 7-fold higher risk of FAS compared to White infants, while infants born in some Southwestern Native American communities have prevalence rates of up to 17.9 per 1000 individuals [11] (US average: 2-7 per 1000 infants) [12]. To date, there is uncertainty regarding the racial disparities in FASDs, as there is a possibility of misestimation of prevalence. Black infants and children are more likely to be misdiagnosed or receive a later diagnosis due to limited access to care, stigmas, or lower socioeconomic status [13]. Contrastingly, concerns have been raised that clinician racial bias results in higher reports of prenatal substance exposure among Black children and lower reports among Hispanic children than White children [13]. Racial and ethnic differences in facial morphology will also result in an overestimation of FASDs among certain African American populations, especially in the absence of a race-specific lip/philtrum guide and 3D evaluations of philtrum height [14].

Advances in technology such as 3D ultrasound allow accurate assessment of dysmorphic facial features among fetuses with FASD [15]. Nevertheless, researchers are questioning whether the current reference standards for facial measurements in assessing FASDs account for such biases and racial/ethnic variations [16]. Current Institute of Medicine (IOM) diagnostic guidelines recommend that ethnic phenotypes should be considered, as what may seem dysmorphic in one racial/ethnic context (ie, lip/philtrum abnormalities or growth retardation, that is, height or weight ≤10th percentile) may be normal in another [17]. Hence, we sought to examine racial/ethnic variations in the prevalence of FASDs using high-quality diagnostic data in which each instance of a FASD was assessed using uniform criteria rather than an administrative assessment (eg, discharge codes on electronic health records).

## Methods

### Study Protocol

As part of a multisite research study of patients with FASD and mothers worldwide conducted since 2003, we analyzed data collected by the Collaborative Initiative on FASDs [18]. We restricted our analyses to sites within the United States, including (1) the Center for Behavioral Teratology at San Diego State University (San Diego, CA); (2) Emory University (Atlanta, GA); (3) 7 Northern Plains communities, including 6 Indian reservations; (4) the University of California, Los Angeles (UCLA; Los Angeles, CA); and (5) the University of Minnesota (Minneapolis, MN) [18]. Methods for obtaining information about prenatal alcohol exposure varied by site and ranged from questionnaires and in-person interviews to reviews of medical, legal, or social service records [18]. For all locations, histories of prenatal alcohol exposure were verified retrospectively [19]. At all sites, the participants were examined for FAS using standardized methodology, as reported by Mattson et al [18,20].

Although the Collaborative Initiative on Fetal Alcohol Spectrum Disorders (CIFASD) also included some women with no alcohol consumption during pregnancy or with unverified prenatal alcohol exposure histories, these samples were not representative of the source populations. Therefore, reliable estimates for average alcoholic content (AAC) could not be calculated. Hence, our analyses were restricted to subjects with at least one incident of verified alcohol consumption during pregnancy. Our objective was to estimate the risk of FASD per gram of pure ethanol intake among women who reported drinking during pregnancy.

### Study Sites

#### Center for Behavioral Teratology Study Site

At this site, children suspected of alcohol exposure were referred to the principal investigator and local professionals for participation in this project [18]. Many patients were already attending this center before the initiation of the CIFASD project, including patients referred to the investigative team for meeting the traditional diagnostic criteria for FAS (eg, facial anomalies, growth retardation, evidence of central nervous system dysfunction (eg, microcephaly, mental retardation, or attentional deficits) [20]. Alcohol exposure histories were obtained via self-reports or professional reviews of medical, legal, or social service records of the biological mother. Parents or primary caregivers completed questionnaires regarding the children’s behavior, while the children were examined for facial features of FAS (ie, 2 of the 3 key facial features: short palpebral fissures [≤10th percentile], a thin vermilion border on the upper lip [rank 4 or 5 on a racially normed lip/philtrum guide], a smooth philtrum [rank 4 or 5 on a racially normed lip/philtrum guide], and signs of pre- or postnatal growth deficiency [head circumference, height, or weight ≤10th percentile]) [19].

#### Emory University Study Site

At Emory University, the Fetal Alcohol and Drug Exposure Clinic gathers data on a large sample of patients with FASDs while providing clinical services and facial evaluations at the Emory University Marcus Institute [21]. In the absence of direct reports, documentation of alcohol abuse or dependence by the biological mother in the form of medical, social services, or court records was reviewed [18]. Recruitment took place via clinical and community referrals. Parents or primary caregivers completed questionnaires and interviews, while patients with FASD were administered various neuropsychological tests over a 3-hour session [22].

#### Northern Plains Study Site

Seven communities, including 1 urban and 6 reservation sites throughout North Dakota, South Dakota, and Montana, participated in this study [21]. Children with FASDs were recruited via active case ascertainment methods and advertisements in tribal and community health centers [19]. Data on prenatal alcohol exposure were obtained from in-person interviews with the parent or primary caregiver to retrospectively obtain exact exposure histories and were also confirmed via reviews of medical records when available [19].

#### University of Minnesota Study Site

The Department of Psychiatry at the University of Minnesota collected data on prenatal alcohol exposure histories through several modalities, including medical reports, birth records, social service records, and, when available, maternal self-reports [21].

#### UCLA Study Site

Data were collected from children attending the Fetal Alcohol and Related Disorders Clinic at UCLA [23]. Participant recruitment was through local FASD clinic referrals, online advertisements, and word of mouth in caregiving communities [23]. All alcohol exposure histories were confirmed via in-person interviews and maternal reports of prenatal substance exposure or reviews of maternal medical records by a licensed medical doctor [23].

### Study Participants

Among children with confirmed prenatal alcohol exposure, a diagnosis of FAS was made if 2 of the 3 key facial features of FAS (ie, short palpebral fissure, smooth philtrum, and thin vermillion border) were accompanied by either microcephaly or growth retardation [18]. Children were excluded when there were reports of known causes of mental deficiency, such as congenital hypothyroidism, neurofibromatosis, or chromosomal abnormalities [18].

### Ethical Considerations

This study was approved by the institutional review board of the Harvard TH Chan School of Public Health (IRB21-1261). Primary data collection at each clinical location of the CIFASD received IRB approval, and informed consent was obtained from all adult participants or their legal guardians [24]. For secondary analysis of the data, our research team was provided with deidentified and anonymized data upon request and approval from CIFASD’s data committee. As a multisite study across several locations and time periods, compensation amount and type varied by collection site; for example, at Emory University, participants were given US $50 as monetary compensation [24,25], while at the University of Southern California and University of Minnesota, participants were compensated for their time with gift cards [26].

### Measures

#### Alcohol Consumption

To calculate the grams of ethanol consumed per day during pregnancy for each pregnant woman, maternal reports of alcohol consumption frequency, quantity, and preferred alcoholic beverage were used. All women were asked to report the frequency (1-2 times during pregnancy, 3-5 times, once/month, 2-3 times/week, almost daily, or daily) and quantity (eg, 2) of alcohol exposure during pregnancy, as well as their preferred alcoholic beverage (eg, beer). Only cases of confirmed exposure were included. Each beverage was converted to grams of ethanol based on average alcoholic content (AAC), that is, 13.6 grams of pure alcohol or one standard drink: 340 mL of regular beer (approximately 5% alcohol), 150 mL of wine (approximately 12% alcohol), and 45 mL of distilled spirits (approximately 40% alcohol) [18]. Upon converting all frequency measures to a daily dose (eg, 1.5/280 days throughout the gestational period, with 1 to 2 incidents of consumption during their entire pregnancy), quantity and AAC were multiplied to determine the grams of pure ethanol consumed by each woman daily.

#### Race/Ethnicity

All participants were asked to self-report their ethnicity as Hispanic/Latino or not Hispanic/Latino. Then, they were asked to self-report their race as American Indian/Alaska Native (AI/AN), Asian, Native Hawaiian or other Pacific Islander, Black/African American, White, more than one race, or other. Owing to the limited number of participants in this investigation for whom there were available data on AAC, they were divided into White, Black, AI/AN, or other.

### Outcome Assessment

At 4 of the sites (excluding the Northern plains), a dysmorphologist was trained to accurately diagnose FAS based on physical features as defined by the CIFASD dysmorphology core [27]. The CIFASD dysmorphology core has been tested in previous studies and has good interrater agreement for height and head circumference, moderate-to-fair agreement for facial anomalies, and poor agreement for the thin vermilion border on the upper lip [28]. Site differences for demographic variables including race, ethnicity, and age group were assessed via a 4-way interaction multivariate analysis of variance (MANOVA) but are not reported, as the effects of demographic variables varied by site [27].

Contrastingly, for the Northern Plains site, a team of physicians, teachers, and other representatives was trained to identify children with certain morphological characteristics of FASDs and other birth defects, as well as IQ and neuropsychologic traits; however, a pediatric dysmorphologist could not be consulted for verification. As “a single generalized set of nonspecific normative physical measures” was often insensitive to local ethnic variations in FASD morphology, syndromic features of FASDs were sometimes compared to normal controls within the same population in terms of weight, head circumference, fissure length, and other facial characteristics (eg, ptosis, and intercanthal distance) [29].

For all CIFASD sites, physical growth and dysmorphology features were recorded on a standardized and weighted checklist, and the IOM criteria for FASDs were used for FAS diagnosis: (1) evidence of a characteristic pattern of minor facial anomalies including at least ≥2 key facial features of FAS (palpebral fissures ≤10th percentile, thin vermilion border, or smooth philtrum), (2) evidence of pre- or postnatal growth retardation (height or weight ≤10th percentile), (3) evidence of deficient brain growth (structural brain anomalies or occipitofrontal circumference ≤10th percentile), and, if possible, (4) confirmation of maternal alcohol consumption directly from the mother or a knowledgeable collateral source [30].

For FAS severity, the IOM-recommended Diagnostic Criteria for FAS and Alcohol-Related Effects were used to determine severity of symptoms in terms of the number of FASD features and type of features (ie, facial anomalies, pre- or postnatal growth deficiency, deficient brain growth, and neurobehavioral impairment) for each patient [30]. FAS was considered the most severe form of FASD, with characteristic facial patterns, growth retardation, and neurodevelopmental abnormalities of the central nervous system (CNS). This was followed by partial FAS, defined as the presence of some components of FAS facial patterns and any of the aforementioned characteristics of FAS [30]; alcohol-related birth defects, which result from congenital defects and malformations or dysplasias of the heart, bone, kidney, vision, or hearing systems; and alcohol-related neurodevelopmental disorders, which include CNS neurodevelopmental abnormalities and complex behavioral and cognitive deficits [30].

Likewise, across all sites, the Child Behavior Checklist (CBCL) was completed by caregivers to collect information regarding child psychopathology and behavioral or emotional functioning [31]. All scores were compiled as a parent-completed survey assessment of their child to determine whether prenatal alcohol exposure affected the child’s ability to internalize or externalize difficult situations; adhere to rules and guidelines; and avoid social, thought, and attention problems [32].

### Statistical Analysis

Descriptive statistics of the overall sample for mean AAC were calculated for participants according to race/ethnicity, maternal age at childbirth, tobacco and substance use (ie, marijuana, amphetamine, cocaine, hallucinogens, heroin, and methadone) during pregnancy, child sex, CBCL score, and clinical location of investigation. To examine the association between race/ethnicity and FAS, as well as FASDs, such as facial anomalies, growth retardation, and deficient brain growth, a log-binomial regression analysis was used while adjusting for child sex, child age, maternal age, and clinic location (as a dummy variable). Analyses were further stratified by race/ethnicity while accounting for a 1-gram unit increase in AAC. All analyses, including the GPLOT SAS procedure for displaying graphs, were performed using SAS software (version 9.4; SAS Institute).

## Results

Table 1 shows the general characteristics of our sample by FAS diagnosis. Overall, 3.4% (20/595) of women who reported consuming alcohol during pregnancy gave birth to a baby with FAS. Women who gave birth to a baby with FAS had a mean AAC of 32.06 (SD 9.09) grams, which was higher than women who did not (mean 12.07, SD 15.87 grams). AI/AN mothers with FAS babies had the highest AAC (mean 42.62, SD 8.35 grams), followed by White (mean 30.13, SD 4.88 grams) and Black mothers (mean 27.05, SD 12.78 grams).

**Figure 1:**
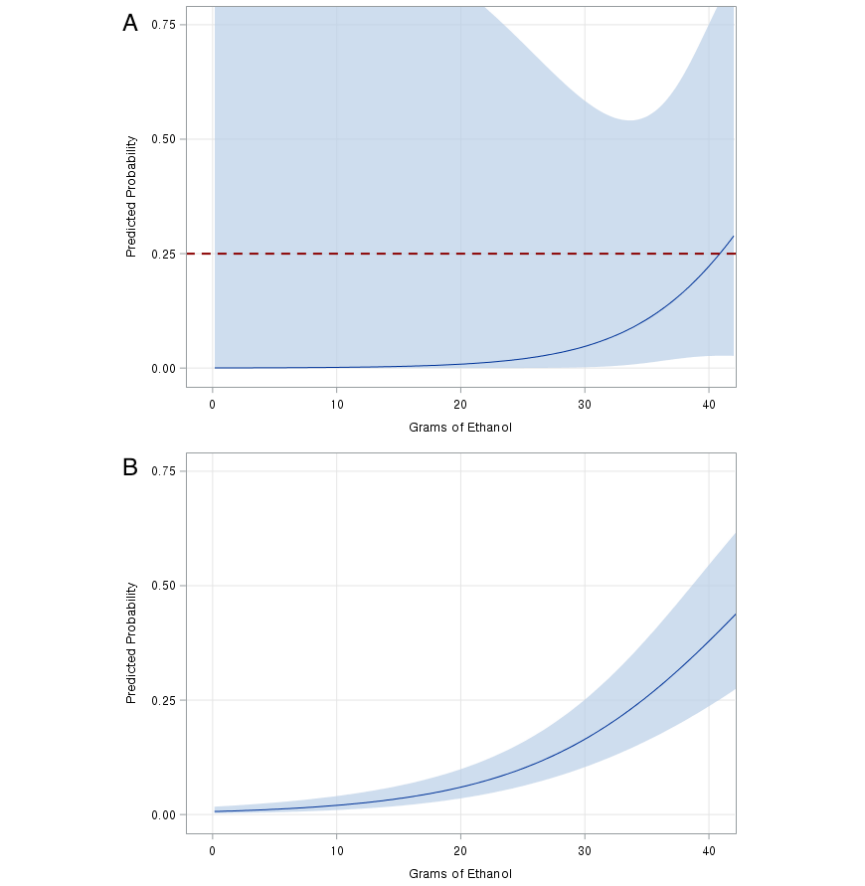
Dose-response effect of average alcoholic content from 0 grams to 45 grams per day on predicted probability of fetal alcohol syndrome. (A) First-trimester drinking only; (B) drinking throughout all trimesters.

As seen in Figure 1, there was a dose-response relationship between AAC and FAS for exposure during different pregnancy trimesters. For women who only consumed alcohol in the first trimester, there was a 25% probability of giving birth to a baby with FAS when they consumed more than 40 grams of pure ethanol (ie, approximately 2.9 standard drinks). Contrastingly, for women who kept drinking alcohol throughout all 3 trimesters of their pregnancy, there was a 25% probability of giving birth to a baby with FAS when they consumed around 30 grams of pure ethanol (ie, approximately 2.1 standard drinks).

**Table 1:** General characteristics of sample by fetal alcohol syndrome diagnosis.

Characteristics			Gave birth to a baby with fetal alcohol syndrome				Did not give birth to a baby with fetal alcohol syndrome		
			Participants, n (%)		AAC^a^ (grams/day), mean (SD)		Participants, n (%)		AAC (grams/day), mean (SD)
**Race**									
	White (n=292)	11 (3.8)		30.13 (4.88)		281 (96.2)		12.29 (15.66)	
	Black (n=254)	4 (1.6)		27.05 (12.78)		250 (98.4)		11.81 (11.55)	
	American Indian/Alaska Native (n=23)	3 (13)		42.62 (8.35)		20 (87)		19.32 (26.13)	
	Other (n=26)	2 (7.7)		37.80^b^		24 (92.3)		10.63 (17.48)	
**Ethnicity**									
	Hispanic or Latino (n=517)	19 (3.7)		32.49 (9.21)		498 (96.3)		12.18 (13.71)	
	Not Hispanic or Latino (n=78)	1 (1.3)		25.20^b^		77 (98.7)		11.13 (15.43)	
**Maternal age at childbirth (years)**									
	<30 (n=297)	9 (3)		31.68 (12.43)		288 (97)		10.87 (13.88)	
	30-35 (n=95)	8 (8.4)		30.86 (5.21)		87 (91.6)		12.21 (15.05)	
	>35 (n=203)	3 (1.5)		37.80^b^		200 (98.5)		13.82 (13.36)	
**Tobacco used during pregnancy**									
	No (n=494)	4 (0.8)		35.20 (4.50)		490 (99.2)		8.60 (11.22)	
	Yes (n=101)	16 (15.8)		29.78 (8.04)		85 (84.2)		18.76 (11.56)	
**Substances used during pregnancy**									
	None (n=514)	9 (1.8)		28.03 (10.01)		505 (98.2)		9.97 (11.47)	
	Marijuana only (n=63)	7 (11.1)		33.60 (6.51)		56 (88.9)		16.96 (12.81)	
	Other (amphetamine, cocaine, hallucinogens, heroin, methadone) (n=18)	4 (22.2)		30.75 (5.22)		14 (77.8)		22.64 (9.86)	
**Child sex**									
	Male (n=331)	8 (2.4)		32.92 (9.51)		323 (97.6)		12.80 (9.71)	
	Female (n=264)	12 (4.6)		31.46 (9.25)		252 (95.5)		10.96 (14.21)	
**CBCL score (points)**									
	Normal (<65) (n=447)	7 (1.6)		36.31 (8.70)		440 (98.4)		9.40 (13.30)	
	Borderline (65-69) (n=65)	6 (9.2)		26.43 (9.94)		59 (90.8)		14.57 (12.52)	
	Clinical (>69) (n=83)	7 (8.4)		33.72 (5.84)		76 (91.6)		18.08 (14.71)	
**Clinical location**									
	San Diego University (n=101)	2 (2)		37.80^b^		99 (98)		17.88 (15.10)	
	Emory University (n=242)	10 (4.1)		31.82 (6.49)		232 (95.9)		10.73 (17.20)	
	Northern Plains (n=51)	5 (9.8)		39.47 (9.29)		46 (90.2)		24.22 (11.26)	
	Minnesota (n=165)	0 (0)		N/A^c^		165 (100)		9.91 (17.05)	
	University of California, Los Angeles (n=36)	3 (8.3)		21.00 (7.27)		33 (91.7)		11.19 (15.06)	
	Total (n=595)	20 (3.4)		32.06 (9.09)		575 (96.6)		12.07 (15.87)	

^a^AAC: average alcoholic content.

^b^SD values missing due to the number of participants being too low.

^c^N/A: not applicable.

As seen in Table 2, AI/AN (PR 9.44, 95% CI 1.78-50.09) mothers and mothers who used tobacco during pregnancy (PR 13.78, 95% CI 4.18-45.35) had significantly increased odds of giving birth to a baby with FAS relative to their counterparts.

As seen in Table 3, when accounting for a 1-gram increase in alcohol consumption during pregnancy, White (PR 1.10, 95% CI 1.03-1.19), Black (PR 1.13, 95% 1.04-1.23), and AI/AN (PR 1.10, 95% CI 1.00-1.21) mothers had 10% to 13% increased odds of giving birth to a baby with FAS given the same exposure to ethanol during pregnancy.

Regardless of race/ethnicity, as seen in Table 4, every 1-gram increase in alcohol consumption during pregnancy was associated with an 11% increased risk of FAS (PR 1.11, 95% CI 1.07-1.15) and 7% increased risk of alcohol-related brain damage (PR 1.07, 95% CI 1.02-1.13). Each 1-gram increase in AAC was also associated with a 4% increase in the chance of giving birth to a baby with ≥2 facial anomalies (ie, short palpebral fissures, thin vermilion border of the upper lip, or a smooth philtrum; PR 1.04, 95% CI 1.02-1.07) or deficient brain growth (PR 1.04, 95% CI 1.01-1.07).

**Table 2:** Log-binomial regression analysis of association between sociodemographic variables and fetal alcohol syndrome, adjusted for child sex, child age, maternal age, and clinical location.

Sociodemographic variables		Prevalence ratio (95% CI)	*P* value
**Race**			
	White (reference)	1.00	
	Black	0.44 (0.14-1.39)	.16
	American Indian/Alaska Native	9.44 (1.78-50.09)	.008
	Other, including Asian, Native Hawaiian or other Pacific Islander, and more than one race	0.40 (0.03-5.52)	.50
**Ethnicity**			
	Hispanic or Latino (reference)	1.00	
	Not Hispanic or Latino	1.94 (0.43-8.76)	.39
**Maternal age at childbirth (years)**			
	<30 (reference)	1.00	
	30-35	2.04 (0.69-6.08)	.20
	>35	0.81 (0.28-2.36)	.69
**Tobacco used during pregnancy**			
	No (reference)	1.00	
	Yes	13.78 (4.18-45.35)	<.001
**Substances used during pregnancy**			
	None (reference)	1.00	
	Marijuana only	1.21 (0.37-3.93)	.75
	Other (amphetamine, cocaine, hallucinogens, heroin, methadone)	3.49 (0.82-14.88)	.09
**Child sex**			
	Male (reference)	1.00	
	Female	3.64 (1.45-9.12)	.006
**Child Behavior Checklist score (points)**			
	Normal (<65) (reference)	1.00	
	Borderline (65-69)	3.32 (1.08-10.19)	.04
	Clinical (>69)	1.89 (0.63-5.69)	.26
**Clinical location**			
	San Diego University (reference)	1.00	
	Emory University	3.19 (0.62-16.44)	.17
	Northern Plains	0.97 (0.17-5.62)	.98
	Minnesota	N/A^a^	N/A
	University of California, Los Angeles	2.26 (0.38-13.58)	.37

^a^N/A: Not applicable (a low number of participants made stratification impossible).

**Table 3:** Log-binomial regression analysis of risk of fetal alcohol syndrome associated with a 1-gram increase in average alcoholic content.

Sociodemographic variables			Prevalence ratio (95% CI)		*P* value
**Race**					
	White	1.10 (1.03-1.19)		.009	
	Black	1.13 (1.04-1.23)		.004	
	American Indian/Alaska Native	1.10 (1.00-1.21)		.05	
	Other, including Asian, Native Hawaiian or other Pacific Islander, and more than one race	N/A^a^		N/A	
**Ethnicity**					
	Hispanic/Latino	1.11 (1.07-1.16)		<.001	
	Not Hispanic/Latino	1.24 (0.02-78.08)		.92	
**Maternal age at childbirth (years)**					
	<30	1.08 (1.02-1.14)		.01	
	30-35	1.04 (0.93-1.17)		.49	
	>35	1.22 (1.07-1.40)		.004	
**Tobacco used during pregnancy**					
	No	1.14 (1.04-1.26)		.006	
	Yes	1.08 (1.04-1.12)		<.001	
**Substances used during pregnancy**					
	None	1.12 (1.01-1.18)		<.001	
	Marijuana only	1.10 (1.02-1.19)		.02	
	Other (amphetamine, cocaine, hallucinogens, heroin, methadone)	1.40 (0.25-7.89)		.70	
**Child sex**					
	Male	1.14 (1.06-1.22)		<.001	
	Female	1.12 (1.05-1.19)		<.001	
**Child Behavior Checklist score (points)**					
	Normal (<65)	1.14 (1.07-1.21)		<.001	
	Borderline (65-69)	2.01 (1.01-4.22)		.05	
	Clinical (>69)	1.10 (1.01-1.20)		.04	
**Clinical location**					
	San Diego University	2.62 (0.39-17.68)		.32	
	Emory University	1.17 (1.09-1.26)		<.001	
	Northern Plains	1.09 (1.01-1.17)		.03	
	Minnesota	N/A^a^		N/A	
	University of California, Los Angeles	0.99 (0.33-2.99)		.99	

^a^N/A: not applicable (a low number of participants made stratification impossible).

**Table 4:** Log-binomial regression analysis of fetal alcohol spectrum disorder severity associated with a 1-gram increase in average alcoholic content.

FASD^a^ characteristics		Participants, n (%)	PR (95% CI)	*P* value
**FASD severity (n=43)**				
	FAS^b^ (most severe)	20 (46.5)	1.11 (1.07-1.15)	<.001
	Partial FAS	1 (2.3)	1.78 (0.59-5.43)	.31
	Alcohol-related neurodevelopmental disorder	9 (20.9)	1.08 (0.96-1.22)	.21
	Alcohol-related birth defects (least severe)	13 (30.2)	1.07 (1.02-1.13)	.008
**Number of FASD features (n=291)**				
	1	209 (71.8)	1.13 (1.04-1.23)	.002
	2	56 (19.2)	1.17 (1.06-1.30)	.002
	3	21 (7.2)	1.38 (0.59-5.43)	.98
	4	5 (1.7)	—^c^	—
**Type of FASD features (n=404)**				
	≥2 Facial anomalies^c^	113 (28)	1.04 (1.02-1.07)	<.001
	Pre- or postnatal growth deficiency	92 (22.8)	1.01 (0.98-1.04)	.52
	Deficient brain growth	51 (12.6)	1.04 (1.01-1.07)	.01
	Neurobehavioral impairment	148 (36.6)	1.00 (0.28-3.53)	.83

^a^FASD: fetal alcohol spectrum disorder.

^b^FAS: fetal alcohol syndrome.

^c^Not available.

^c^Facial anomalies included short palpebral fissures (≤10th percentile), a thin vermilion border on the upper lip (rank 4 or 5 on a racially normed lip/philtrum guide) and a smooth philtrum (rank 4 or 5 on a racially normed lip/philtrum guide).

## Discussion

### Principal Results

Our results suggest that although the average quantity of alcohol consumed during pregnancy varied by race/ethnicity, the risk of delivering a baby with FAS or FASDs did not vary by more than 3% among White, Black, and AI/AN women when equal doses of ethanol were consumed. There is a consensus that “for unclear reasons, there is an increased risk of FAS in those who belong to African American or Native American ethnicities” [33]. Some researchers have attributed this to genetic polymorphisms that result in an increased risk of FAS among certain races [34,35]. African American race/ethnicity is believed to increase FAS risk due to the ADH1B*3 functional polymorphism—found in approximately 33% of those with African ancestry and almost exclusively in this group, which has a 70-to-80-time higher conversion rate of ethanol to acetaldehyde [36]. However, a previous study attributed to the maternal CIF*3 allele found among African Americans a protective effect against adverse prenatal effects of alcohol [37]. The high prevalence of FAS in certain communities (eg, the Indigenous) is likely due to more intensive screening or other social and environmental risk factors (eg, lead exposure or poor diet) than innate racial characteristics [38].

Our findings also suggest that innate racial characteristics are likely unassociated with the risk of FASDs for any race, especially when AAC is controlled for. Regarding Hispanic/Latino women, our study also found no statistically significant association between ethnicity and risk of FAS when AAC was accounted for, and this was in alignment with a previous study reporting that the pattern of early-pregnancy alcohol consumption in Latinas of childbearing age residing in the United States was similar to that of other racial/ethnic groups [39].

As data on patients with FAS or FASDs and their exposure histories have remained sparse [40], it was difficult to adjust for AAC accurately before the public release of the CIFASD data. While existing nationally representative data sets, such as the National Birth Defects Prevention Study (NBDPS) [41] and Pregnancy Risk Assessment Monitoring System (PRAMS) [42] have some information on women’s health-related behaviors during pregnancy, they do not have a detailed assessment of drinking behaviors, such as the exact type of alcoholic beverage consumed (eg, beer, wine, or distilled spirits) [43], which makes it impossible to calculate exact AACs.

### Limitations

This study has limitations. Firstly, all data were self-reported; many studies have commented on the underreporting of alcohol consumption during pregnancy by mothers [44,45]. Our calculations of AAC need to be interpreted with caution, as the retrospective manner in which study participants were interviewed made it difficult to collect exact, day-to-day measurements of the quantity, frequency, and type of alcoholic beverage consumed.

As mentioned above, our sample size was also limited and not nationally representative of the United States, which may have resulted in sampling bias owing to the clinical locations where patients were recruited for the CIFASD project. This is likely the reason why the prevalence of pregnant drinkers among certain races and ethnicities in our sample differed from that of previous studies that used nationally representative data sets, such as the NBDPS or PRAMS [42]. Due to the manner in which the CIFASD collected data at certain sites (eg, word of mouth, advertisements in tribal and community health centers, and existing patients at referral clinics), the data on the prevalence of FAS and pregnant drinkers and nondrinkers by race or ethnicity presented in Table 1 should not be interpreted as representative of the general United States or AI/AN population. Among AI/AN people, FASDs are widely recognized as a “major public health priority” [46], since higher patterns of alcohol consumption result in a higher prevalence of FASDs compared to the general population [47].

The small number of FAS-diagnosed individuals (n=2) in the AI/AN sample (n=23) is reflected by the highly imprecise PR estimate for AI/AN individuals (PR 9.44, 95% CI 1.78-50.09). In the United States, AI/AN individuals are only around 2% of the population (for a total of approximately 5.2 million people) [48], and only 14 states (including New Mexico, where the CIFASD collected data for this study) have more than 100,000 AI/AN residents [49]. FAS remains a major concern for the AI/AN community, and previous studies have noted that AI individuals have 3 times the rate of fetal alcohol births [50], possibly because of polymorphisms related to aldehyde dehydrogenase and acetaldehyde dehydrogenase genes found in AI populations [51].

However, until more data are collected for this community, we believe that caution is warranted in ascribing racial disparities in FAS diagnosis to genetic or biological susceptibility [51]. For example, the high prevalence of alcohol use disorders in AI/AN groups has been shown to be driven by primarily nonbiological factors [52].

Furthermore, previous studies have highlighted the importance of considering immigration history and its interaction with race and ethnicity [53]. Trends in child health in the United States, especially with regard to Hispanic- or Asian-origin populations, may be influenced by immigration status and extent of acculturation [53]; for example, relative to United States–born women, it is believed that non–United States–born women are less likely to initiate early prenatal care (PNC) or receive adequate PNC due to lower rates of insurance coverage and patient-provider communication gaps [54]. The CIFASD data set does not include information on maternal immigration or health insurance status, and we encourage future studies to explore the interaction between such factors and FAS onset, as mothers of children who have FAS are more likely to be without PNC, be on Medicaid at childbirth, and potentially have different health-related behaviors, depending on naturalization status [55].

Because of the small sample size of individuals with FAS at different clinical locations, as seen in certain parts of our analyses (ie, the log-binomial analysis of FAS individuals from Minnesota University [n=0] and the subgroup analysis of other races [n=2] for FAS onset associated with a 1-gram increase in AAC), some PRs could not be calculated. In future studies with larger sample sizes, we hope that there will be more information on FAS incidence, so that quantitative analyses with various stratifications of variables are possible.

### Conclusions

The current IOM guidelines for FASD diagnosis recommend that racial/ethnic variations in the expression of key features of FASDs be carefully examined, if possible, for the detection of minor physical variations [56]. However, more research that takes into consideration drinking behaviors and cultural drinking norms among each racial/ethnic group in the United States is warranted to determine whether the onset of FAS and related disabilities is biological or behavioral.

## Abbreviations

AAC: average alcoholic content
AI/AN: American Indian/Alaska Native
CBCL: Child Behavior Checklist
CIFASD: Collaborative Initiative on Fetal Alcohol Spectrum Disorders
FAS: fetal alcohol syndrome
FASD: fetal alcohol spectrum disorder
IOM: Institute of Medicine
NBDPS: National Birth Defects Prevention Study
PNC: prenatal care
PR: prevalence ratio
PRAMS: Pregnancy Risk Assessment Monitoring System
UCLA: University of California, Los Angeles

## Data Availability

Our agreement with the Collaborative Initiative on Fetal Alcohol Spectrum Disorder for accessing the CIFASD database does not include authorization of access to a third party. Request for database access should be addressed to CIFASD’s data committee [57].
