# Correction: Racial/Ethnic Disparity in Association Between Fetal Alcohol Syndrome and Alcohol Intake During Pregnancy: Multisite Retrospective Cohort Study

**DOI:** 10.2196/48460

**Published:** 2023-04-28

**Authors:** Sarah Soyeon Oh, Bada Kang, Jewel Park, SangMin Kim, Eun-Cheol Park, Seung Hee Lee, Ichiro Kawachi

**Affiliations:** 1 Department of Social and Behavioral Sciences Harvard TH Chan School of Public Health Boston, MA United States; 2 Institute of Health Services Research Yonsei University College of Medicine Seoul Republic of Korea; 3 Mo-Im Kim Nursing Research Institute Yonsei University College of Nursing Seoul Republic of Korea; 4 Department of Pediatrics Cincinnati Children’s Hospital Medical Center Cincinnati, OH United States; 5 Harvard Medical School Boston, MA United States; 6 Department of Preventive Medicine and Public Health Yonsei University College of Medicine Seoul Republic of Korea; 7 College of Nursing and Brain Korea 21 Four Project Yonsei University Seoul Republic of Korea

In “Racial/Ethnic Disparity in Association Between Fetal Alcohol Syndrome and Alcohol Intake During Pregnancy: Multisite Retrospective Cohort Study” (JMIR Public Health Surveill 2023;9:e45358) the authors noted one error.

The authorship was previously published as:

Sarah Soyeon Oh, SangMin Kim, Bada Kang, Jewel Park, Seung Hee Lee, Eun-Cheol Park, Ichiro Kawachi

And will be changed to read as follows:

Sarah Soyeon Oh, Bada Kang, Jewel Park, SangMin Kim, Eun-Cheol Park, Seung Hee Lee, Ichiro Kawachi

The correction will appear in the online version of the paper on the JMIR Publications website on April 28, 2023 together with the publication of this correction notice. Because this was made after submission to PubMed, PubMed Central, and other full-text repositories, the corrected article has also been resubmitted to those repositories.

